# Species' Life-History Traits Explain Interspecific Variation in Reservoir Competence: A Possible Mechanism Underlying the Dilution Effect

**DOI:** 10.1371/journal.pone.0054341

**Published:** 2013-01-24

**Authors:** Zheng Y. X. Huang, Willem F. de Boer, Frank van Langevelde, Valerie Olson, Tim M. Blackburn, Herbert H. T. Prins

**Affiliations:** 1 Resource Ecology Group, Wageningen University, Wageningen, The Netherlands; 2 Department of Biology and Biochemistry, University of Bath, Bath, United Kingdom; 3 Institute of Zoology, Zoological Society of London, London, United Kingdom; 4 Distinguished Scientist Fellowship Program, King Saud University, Riyadh, Saudi Arabia; Metabiota, United States of America

## Abstract

Hosts species for multi-host pathogens show considerable variation in the species' reservoir competence, which is usually used to measure species' potential to maintain and transmit these pathogens. Although accumulating research has proposed a trade-off between life-history strategies and immune defences, only a few studies extended this to host species' reservoir competence. Using a phylogenetic comparative approach, we studied the relationships between some species' life-history traits and reservoir competence in three emerging infectious vector-borne disease systems, namely Lyme disease, West Nile Encephalitis (WNE) and Eastern Equine Encephalitis (EEE). The results showed that interspecific variation in reservoir competence could be partly explained by the species' life histories. Species with larger body mass (for hosts of Lyme disease and WNE) or smaller clutch size (for hosts of EEE) had a higher reservoir competence. Given that both larger body mass and smaller clutch size were linked to higher extinction risk of local populations, our study suggests that with decreasing biodiversity, species with a higher reservoir competence are more likely to remain in the community, and thereby increase the risk of transmitting these pathogens, which might be a possible mechanism underlying the dilution effect.

## Introduction

Diseases caused by multi-host pathogens are able to impact livestock productivity, agricultural economies, wildlife conservation and public health [1]. For many infectious multi-host pathogens, different host species, or even co-occurring host species in the same community, exhibit pronounced variation in their abilities to serve as reservoirs or transmit the pathogens [2], [3]. Therefore, it is a major concern to better understand the dynamics of disease transmission, especially at community level, and the impact of differences in reservoir competence on infection risk [2].

Reservoir competence is usually used to measure a species' potential to serve as a reservoir for pathogens and transmit pathogens [4]–[6]. Recently, ecologists have begun to search for explanations for the interspecific variation in reservoir competence in the ecology and life histories of species [2], [7]. Life history theory generally suggests trade-offs with investment in self-maintenance (e.g., physiological resistance) at the expense of other physiological activities, such as current reproduction and growth [8]. The predictions derived from this theory suggest that “fast-lived” species (i.e. species that follow a strategy aimed at growth and early reproduction) tend to invest minimally in adaptive immunity [9], [10], which may make them more competent for pathogens [11], whereas “slow-lived” species with longer life spans and slower growth rates are hypothesized to invest more into costly immune defences. Several studies have shown that specific immune defence level could be related to life-history traits, such as fecundity [12] and developmental period [9]. However, only a few studies extended this trait-based approach to examine the relationships between the hosts' life-history traits and the potential to transmit pathogens (but see Cronin et al. [2]). Better understanding these relationships could help us to predict the species' reservoir competence and model disease dynamics at community level, which is relevant for human health, economic growth and wildlife conservation [1], [2], [7].

In this paper, we present a quantitative study relating life-history traits to the variation in species' reservoir competence for three vector-borne diseases: one tick-borne disease, Lyme disease and two mosquito transmitted diseases, West Nile Encephalitis (WNE) and Eastern Equine Encephalitis (EEE). We used the reservoir competence index (RCI) as a measure of the species' reservoir competence, which is considered to be a function of several epidemiological parameters, namely host susceptibility (probability of a host becoming infected by infected vectors), host infectivity (probability of a vector becoming infected, when feeding on an infected host), and duration of infectiousness (number of days that a host remains infectious) [2], [4], [6], [13]. For species life-history traits, we used body mass, incubation time (gestation time for mammals), and clutch size (litter size for mammals). Incubation time and clutch size have been linked to the species' immune response [9], while body mass can serve as a surrogate for size-scaled life-history traits such as fecundity, metabolic requirements [14] and age at first breeding [15].

In addition, a species' potential to serve as a reservoir or transmit pathogens may have a phylogenetic signal. Since the morphological and physiological traits of species which regulate interactions with pathogens are usually phylogenetically conserved [16], phylogenetic differences in reservoir competence may exist across different taxa [3]. Therefore, we use both a conventional and a phylogenetic comparative analysis to test the relationships between the life-history traits and reservoir competence. We expect reservoir competence to be negatively correlated with body mass and incubation time (gestation time for mammals) while positively correlated with clutch size (litter size for mammals).

## Materials and Methods

### (a) Data collection

We searched for reservoir competence data from published studies and found reservoir competence data for three vector-borne diseases (Table 1). For Lyme disease, we collected the data from studies about *Borrelia* and different tick vector species. Since different strains of pathogens and different tick vector species may influence host reservoir competence [17], we only used the data from those studies where the disease is caused by the etiologic agent *Borrelia burgdorferi* and transmitted by the vector *Ixodes scapularis* [4], while the numbers of host species in the data sets with respect to other strains of *Borrelia* or other tick vector species were too small. For Lyme disease, we used the species' realized reservoir competence (RRC), i.e. the product of the species' host susceptibility and host infectivity, as a measure for the species' reservoir competence [4] because of the lack of data on the duration of infectiousness. For WNE, we used two different data sets (Table 1, two data sets are referred as WNE-1 and WNE-2 respectively): the first data set determined the reservoir competence index and host infectivity for 25 native bird species of North America in experimental conditions [6], whereas the second described original raw experimental viremia data from different studies and recalculated the reservoir competence index for 44 bird species using a method to avoid inflation of average viremia and infectiousness by a single animal with a high-titred viremia [18]. For EEE, we used the published dataset of 10 bird species [5].

**Table 1:** Disease parameters, studied taxon, number of host species used in the analysis of Lyme disease, West Nile Encephalitis (WNE) and Eastern Equine Encephalitis (EEE).

Disease	Host taxon	Disease parameter	Host number
Lyme disease	mammal	realized reservoir competence (RRC)	9
WNE-1	bird	reservoir competence index (RCI)	15
WNE-2	bird	reservoir competence index (RCI)	24
EEE	bird	reservoir competence index (RCI)	10

We collected life-history traits data (body mass, gestation/incubation time and litter/clutch size) from previous published studies or existing databases. Data sources are listed in Table S1 and Table S2.

### (b) Phylogenetic tree

For WNE and EEE, we used a published phylogenetic tree of birds [19], which includes 169 avian and 2 out-group genera. If only one bird species in the disease data set did belong to a genus in the tree, the genus tip was considered as the tip of this species. If more than one bird species did belong to a genus in the tree, we added a new branch with length 0.0001 for each species to the genus tip, and then the genus tip became a node. For the bird species which did not belong to any genus in this phylogenetic tree, we checked if the tree included any genera sharing the same family with these bird species. Species which did not belong to any family derived from the genera in the tree were not used in the analysis. If there was only one genus in the tree sharing the same family with the bird species in the disease data, the genus tip was considered as the tip for this species. If a bird species shared the same family with more than one genus in the tree, we created a new ‘family’ tip [20]. Then this ‘family tip’ was used as the tip of the bird species in the disease dataset. For Lyme disease, we used a published phylogenetic tree including almost all extant mammal species [21]. Trees were transformed to ultrametric trees (Figure S1 and Figure S2) to perform the phylogenetic comparative analysis.

### (c) Statistical analysis

In the datasets of WNE used in the study, there were several non-host bird species whose reservoir competences were zero. Non-hosts data were removed before analysis because within a community there are many non-host species which are often not included in reservoir competence studies, especially for the studies with respect to testing life history theory, since trade-offs between life-history traits versus immune defence against a specific pathogen might not occur in non-host species.

We log-transformed incubation time (gestation time for mammals) and body mass. We fitted models using reservoir competence as dependent variable and life-history traits as independent variables. We reported the results of a non-phylogenetic statistical analysis (assuming a star phylogeny [20]), and a phylogenetic comparative analysis under Brownian motion evolution. Since life-history traits were usually significantly correlated with each other and the relationship of a trait might be changed by adding other collinear variables in multiple regression models, we first conducted a factor analysis to extract the primary life-history axes, and reported the results of the univariate regressions using these extracted factor scores as independent variables. For Lyme disease, we first conducted our analyses using phylogenetic independent contrasts for all variables, then extracted the primary life-history axes from these independent contrasts, and finally carried out regression analyses on these phylogenetically corrected responses and predictors [7]. For WNE and EEE, since the phylogenetic tree of birds was not fully dichotomous because of the lack of some branches' lengths (Figure S2), we first conducted the factor analyses and then carried out the regression analyses using a phylogenetic GLS approach instead of the independent contrast approach [22]. After that, we also carried out univariate regression analyses to test for the impact of each life-history trait on the species' reservoir competence. All analyses were carried out in Canoco 5 and R 2·14·0 using the *ape* package [23].

## Results

### (a) Factor analysis

**Figure 1:**
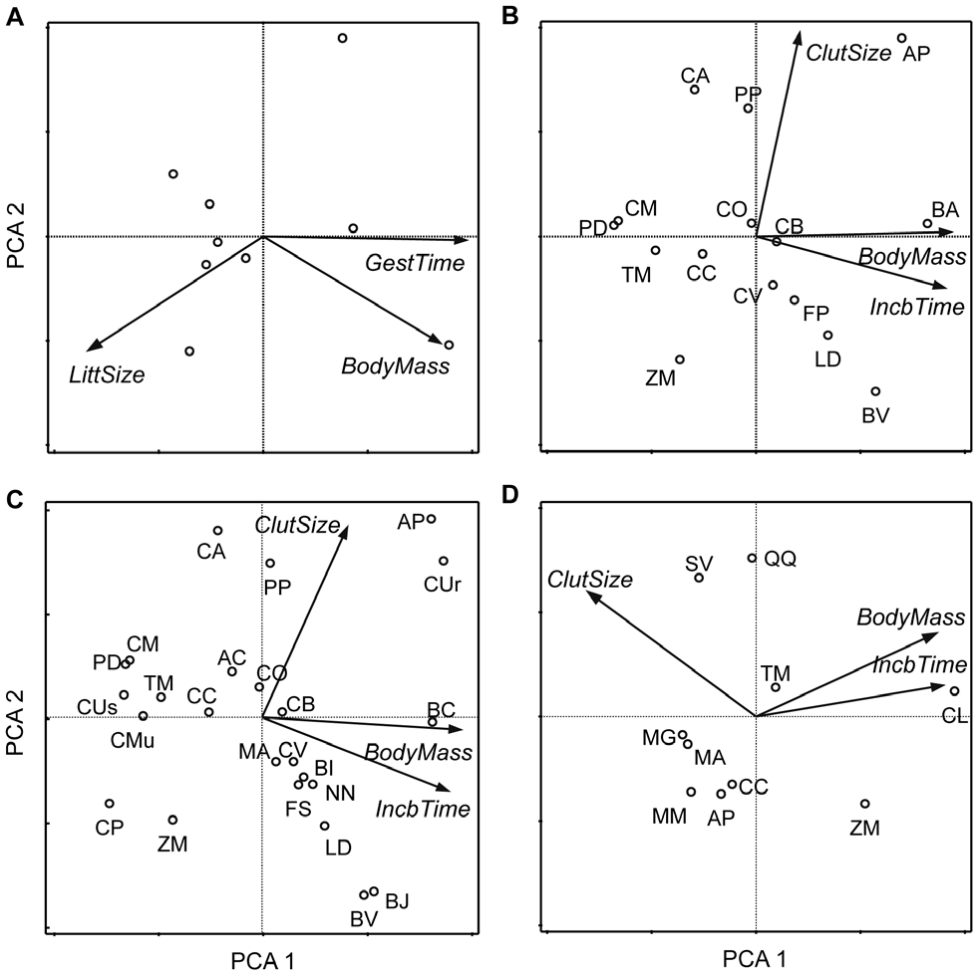
The results of factor analyses for the life-history traits of host species. A. Factor analysis for mammal hosts of Lyme disease. B. Factor analysis for bird hosts used in WNE-1. C. Factor analysis for bird hosts used in WNE-2. D. Factor analysis for bird hosts of EEE. Species codes plotted in ordination space reflect the first two letters of the genus and species names (for Lyme disease, the species codes cannot be given because the species' names of the internal nodes were not available).

Factor analyses (Figure 1) showed that the first component axis, Factor 1, explained a large percentage of the variance of the species' life-history traits: 78.5% for the hosts of Lyme disease, 57.2% for the hosts of WNE-1, 61.8% for the hosts of WNE-2 and 72.1% for the hosts of EEE. For Lyme disease and EEE, all three life-history traits were heavily loaded on Factor 1. Whereas for WNE-1 and WNE-2, only body mass and incubation time were heavily loaded on Factor 1, and clutch size was generally more extracted on the second Factor. Host species with higher Factor 1 scores were generally those that have “slow-lived” characteristics, e.g. larger body mass, longer incubation/gestation time and smaller litter/clutch size (only in Lyme disease and EEE).

### (b) Regression analysis

The phylogenetic regression analyses of Factor 1 (Table 2) showed that the realized reservoir competence of Lyme disease, reservoir competence index in WNE-1 and EEE were all significantly negatively correlated to the Factor 1 scores. According to these results, higher Factor 1 scores referred to slower life histories, those species with higher reservoir competence tended to have fast life histories. The reservoir competence index in WNE-2 was not significantly associated to the Factor 1 scores (Table 2).

**Table 2:** Regression coefficient *b*, *t*-statistic and adjusted *R^2^* (only for conventional analysis) for the univariate linear regressions of the first primary component (Factor 1) for both non-phylogenetic and phylogenetic analysis of Lyme disease, West Nile Encephalitis (WNE) and Eastern Equine Encephalitis (EEE).

		Conventional analysis			Phylogenetic analysis	
Disease, data resource	Disease parameters	*b*	*t*	Adjusted *R^2^*	*b*	*t*
Lyme disease (*n* = 9)	RRC#	−0.10	−1.48	0.13	−0.14	−2.72*
WNE-1 (*n* = 15)	RCI#	−0.21	−1.63	0.11	−1.22	−2.97*
WNE-2 (*n* = 24)	RCI	−0.02	−0.21	−0.04	−0.48	−1.16
EEE (*n* = 10)	RCI	−0.23	−1.98	0.24	−0.17	−2.71*

# RRC: realized reservoir competence. RCI: reservoir competence index.

* *p*≤0.05,

**p≤0.01.

In regression analyses for each life-history trait, both non-phylogenetic and phylogenetic analysis showed that body mass was the strongest predictor for the species' realized reservoir competence of Lyme disease (Table 3). Species with a larger body mass tended to have a lower realized reservoir competence for Lyme disease. Neither gestation period nor litter size showed any significant relationship with realized reservoir competence, though the coefficients were, as expected, negative for gestation and positive for litter size (Table 3).

**Table 3:** Regression coefficient *b*, *t*-statistic and adjusted *R^2^* (only for conventional analysis) for the univariate linear regressions of each life-history traits for both non-phylogenetic and phylogenetic analysis of Lyme disease, West Nile Encephalitis (WNE) and Eastern Equine Encephalitis (EEE).

			Conventional analysis			Phylogenetic analysis	
Disease, data resource	Disease parameters	Independent variables	*b*	*t*	Adjusted *R^2^*	*b*	*t*
Lyme disease (*n* = 9)	RRC#	body mass	−0.18	−3.68**	0.61	−0.19	−3.55**
		gestation	−0.17	−1.32	0.08	−0.16	−1.23
		litter size	0.01	0.13	−0.14	0.01	0.24
WNE-1 (*n* = 15)	RCI#	body mass	−0.49	−1.75	0.11	−1.54	−2.68*
		incubation	−0.36	−0.74	−0.03	2.64	0.40
		clutch size	−0.09	−0.81	−0.02	−0.36	−2.15
WNE-2 (*n* = 24)	RCI	body mass	−0.01	−0.04	−0.03	−0.45	−0.87
		incubation	0.17	0.53	−0.02	13.57	1.44
		clutch size	0.06	0.80	−0.01	−0.29	−1.63
EEE (*n* = 10)	RCI	body mass	−0.52	−1.02	0.01	−0.31	−0.78
		incubation	−1.87	−1.17	0.04	−0.25	−0.59
		clutch size	0.42	3.32*	0.53	0.37	2.92*

# RRC: realized reservoir competence. RCI: reservoir competence index.

* *p*≤0.05,

** *p*≤0.01.

For species' reservoir competence index of WNE-1, the phylogenetically corrected univariate regression showed significantly negative relationships with body mass (Table 3). Species with a larger body mass tended to have lower reservoir competence index for WNE. Whereas for the second WNE data set (WNE-2), no significant relationships between reservoir competence and life-history traits were found in the non-phylogenetic regression or in the phylogenetic regression (Table 3).

For EEE, both the results of the non-phylogenetic univariate regression and phylogenetic analysis showed that clutch size was a significant predictor for species' reservoir competence index (Table 3). Species with larger clutch size tend to have a higher reservoir competence for EEE. Neither body mass nor incubation time showed any significant relationships with reservoir competence, though the coefficients were, as expected, negative (Table 3).

## Discussion

Our study focused on the relationships between life-history traits and species' reservoir competence for three vector-borne diseases. The results generally showed that life-history traits can partly explain interspecific variation in reservoir competence. Body mass is a strong predictor to the reservoir competence in Lyme disease and WNE-1. Larger-bodied species tend to have lower reservoir competence. The variation in birds' reservoir competence in EEE could be partly explained by clutch size. As we predicted, bird species with larger clutches tend to have a higher reservoir competence of EEE. For reservoir competence index in WNE-2, the lack of a significant relationship might be due to the different sources used in compiling this data set. The reservoir competence index can differ when measured under different conditions, since one component of reservoir competence index, the species' susceptibility, usually vary in space and over time [4].

Our findings build on an emerging body of studies on the relationships between life history theory and disease ecology. Instead of focusing on immunology, however, our study associated the species' potential to maintain and transmit pathogens with life-history traits. Life history theory suggests the existence of a trade-off between the immune system and life-history traits relating to growth and reproduction [24], [25]. “Slow-lived” species tend to invest more in adaptive immunity because they probably encounter a greater number of infections overall, and are more likely to encounter the same pathogen, whereas “fast-lived” species which are in favour of growth and frequent reproduction tend to invest comparatively little in costly adaptive immunity [10], [24]. Together with a previous study suggesting that species with a higher reservoir competence tend to favour cheaper, nonspecific immune defences that pathogens may be able to circumvent easily [26], the negative relationships between reservoir competence and life histories in our study support the predictions derived from life history theory. In addition, previous studies reported a strong positive relationship between natural antibody levels and incubation period in bird and mammal species [9], [26], indicating that longer developmental times contribute to better adaptive immune systems. However, we did not find any significant relationship between incubation/gestation time and reservoir competence. This indicates that other factors, besides the effect of incubation period on adaptive immune system, might also influence species' reservoir competence, which needs to be studied in the future.

Recently several studies on life history theory proposed to discuss these physiological trade-offs between defence versus life histories in the context of a broader background, namely, the impact of biodiversity on disease transmission [2], [7], [26]. Based on our results, one might expect that those species with a high reservoir competence are more likely to be those that are wide-distributed, since evidence is accumulating that species with faster life histories are more resistant to population decline and local extinction than “slow-lived” species [14], [27]. Species with faster life histories (such as those with smaller body masses and larger clutch sizes) usually have lower energetic requirements and higher reproductive capacities, which make them more likely to be able to survive in remnant habitat patches with low biodiversity [27]. Also, some studies suggested that larger body mass usually associated with smaller population size [28], which also make them more vulnerable to biodiversity decline [29]. According to our findings that the species' reservoir competence can be partly explained by their life histories, species with slower life histories tend to have lower reservoir competence. Thus, the species which are first lost from a community when disturbed tend to be those that are less competent hosts, ultimately leaving a higher abundance of more competent species in low diversity systems due to release from competition or predation, and thereby increase the risk for disease transmission. This might be a possible mechanism underlying the dilution effect, the inverse relationship between biodiversity and disease risk, which has attracted much interest in the context of ongoing biodiversity losses and increased emergence of human and wildlife diseases [11], [30], [31].

In theory, high biodiversity might dilute or amplify disease risk by changing the relative abundance of competent hosts [32]. The amplification effect suggests a positive relationship between biodiversity and disease risk. Compared with rare studies that support the amplification effect [11], the dilution effect has been reported for quite a few different diseases [13], [33]–[40]. Some studies have shown that the dilution effect generally occurs when competent host species survive and increase their local densities in disturbed low-diversity communities, while other ecologists criticised the dilution effect and claimed that the dilution effect only occurs under certain circumstances and depends on a specific community composition where incompetent host species are more likely to be present in high-diversity communities [41]. Together with a previous study which suggested that fast-lived amphibian species were particularly prone to infection and pathology of a virulent trematode parasite, *Ribeiroia ondatrae* [7], the results of our study might explain how the community composition changes under increasing species loss and how this affects the species' competence for the pathogen, triggering a dilution effect.

Our study highlights the importance of the association between life-history traits and species' potential to reserve and transmit pathogens and thus contributes to empirical evidence for life history theory. The results, in conjunction with findings of relationships between species' life histories and local extinction risk, suggest a possible mechanism why the dilution effect operates with decreases in biodiversity.

## Supporting Information

Figure S1 **Phylogenetic tree of mammals used for Lyme disease.**
(TIFF)

Figure S2 **Phylogenetic tree of birds used for WNE and EEE.**
(TIFF)

Table S1 **Data sources for reservoir competence, mammals' life-history traits and birds' body mass.**
(DOCX)

Table S2 **Body mass, clutch size and incubation period of birds used in the analysis.**
(DOCX)
