# Correction: Species' Life-History Traits Explain Interspecific Variation in Reservoir Competence: A Possible Mechanism Underlying the Dilution Effect

**DOI:** 10.1371/annotation/edc86621-7e9e-4702-b5d3-40cf19ebf731

**Published:** 2013-09-13

**Authors:** Zheng Y. X. Huang, Willem F. de Boer, Frank van Langevelde, Valerie Olson, Tim M. Blackburn, Herbert H. T. Prins

There was an error in the penultimate sentence of the Abstract. The correct sentence is: Species with larger body mass (for hosts of Lyme disease and WNE) or smaller clutch size (for hosts of EEE) had a lower reservoir competence. 

