# Supplementary material for: Species' Life-History Traits Explain Interspecific Variation in Reservoir Competence: A Possible Mechanism Underlying the Dilution Effect
Source: PLoS One. 2013 Jan 24;8(1):e54341. doi: 10.1371/journal.pone.0054341 (PMC3554779; doi:10.1371/journal.pone.0054341)
Supplement: Table S1 — Data sources for reservoir competence, mammals' life-history traits and birds' body mass. (DOCX) [file pone.0054341.s003.docx]

**Table S1:** Data sources for reservoir competence, mammals’ life-history traits and birds’ body mass

| variables | sources or references |
| --- | --- |
| RRC for Lyme disease | LoGiudice K et al. 2003 (Table 1) |
| RCI for WNE-1 | Komar N et al. 2003 (Table 10) |
| RCI for WNE-2 | Kilpatrick AM. 2007 (Figure 2) |
| RCI for EEE | Komar N et al. 1999 (Table 3) |
| life-history traits for mammals | Jones KE. *Ecological Archives* E090-184-D1 |
| body mass for birds | Olson et al. 2009 |
