# Supplementary material for: Species' Life-History Traits Explain Interspecific Variation in Reservoir Competence: A Possible Mechanism Underlying the Dilution Effect
Source: PLoS One. 2013 Jan 24;8(1):e54341. doi: 10.1371/journal.pone.0054341 (PMC3554779; doi:10.1371/journal.pone.0054341)
Supplement: Table S2 — Body mass, clutch size and incubation period of birds used in the analysis. (DOCX) [file pone.0054341.s004.docx]

**Table S2:** Body mass, clutch size and incubation period of birds used in the analysis

| **species** | **body mass (g)** | **clutch size** | **incubation period (day)** | **sources and references** |
| --- | --- | --- | --- | --- |
| Agelaius phoeniceus | 53.22 | 3.28 | 11.68 | Bennett PM 2002 |
| Agelaius tricolor | 66.53 | 3.75 | 11.875 | Bennett PM 2002 |
| Anas platyrhynchos | 1096.33 | 8.25 | 27.5 | Bennett PM 2002;  del Hoyo J et al. 1994 |
| Aphelocoma californica | 85.38 | 4.8 | 18.2 | Martin TE 1995 |
| Bubo virginianus | 1205.96 | 2.25 | 30.15 | Bennett PM 2002 |
| Bubulcus ibis | 345.43 | 3.67 | 23.85 | Bennett PM 2002;  Brown LH et al. 1982 |
| Buteo jamaicensis | 1121.90 | 2.43 | 32 | Bennett PM 2002;  del Hoyo J et al. 1994 |
| Cardinalis cardinalis | 44.70 | 3.37 | 12.5 | Bennett PM 2002 |
| Carpodacus mexicanus | 21.39 | 4.33 | 13.53 | Bennett PM 2002 |
| Catharus mustelinus | 49.65 | 3.43 | 13.23 | Bennett PM 2002 |
| Catharus ustulatus | 31.19 | 3.66 | 12.5 | Bennett PM 2002 |
| Centrocercus urophasianus | 2722.81 | 7.5 | 26 | Bennett PM 2002 |
| Charadrius vociferus | 92.42 | 4 | 26.57 | Bennett PM 2002 |
| Colaptes auratus | 136.21 | 6.47 | 12.625 | Bennett PM 2002 |
| Columba livia | 332.47 | 2 | 17.25 | Bennett PM 2002 |
| Columbina passerina | 30.29 | 2 | 13.5 | Bennett PM 2002 |
| Corvus brachyrhynchos | 445.27 | 4.25 | 18.15 | Bennett PM 2002 |
| Corvus ossifragus | 284.26 | 4.5 | 17 | Bennett PM 2002 |
| Cyanocitta cristata | 74.56 | 4 | 17 | Bennett PM 2002 |
| Dumetella carolinensis | 37.08 | 3.88 | 13.3 | Bennett PM 2002 |
| Euphagus cyanocephalus | 63.19 | 5.09 | 12.75 | Bennett PM 2002;  Martin SG 2002 |
| Falco sparverius | 116.52 | 3.85 | 29.25 | Bennett PM 2002 |
| Larus delawarensis | 517.06 | 3.07 | 26.1 | Bennett PM 2002 |
| Melospiza melodia | 20.68 | 3.87 | 12.7 | Bennett PM 2002;  Arcese P et al. 2002 |
| Mimus polyglottos | 48.71 | 3.90 | 12.32 | Bennett PM 2002 |
| Molothrus ater | 43.21 | 4 | 11.57 | Bennett PM 2002;  Lowther PE 1993 |
| Nycticorax nycticorax | 550.42 | 3.50 | 23 | Bennett PM 2002 |
| Megascops asio | 184.50 | 4 | 26 | Bennett PM 2002 |
| Passer domesticus | 26.98 | 4.15 | 12.51 | Bennett PM 2002 |
| Pica pica sericea | 225.50 | 6.5 | 17 | Madge S & Burn H 1999 |
| Quiscalus quiscula | 116.02 | 4.83 | 13.67 | Bennett PM 2002 |
| Sturnus vulgaris | 81.70 | 5.12 | 12.375 | Bennett PM 2002 |
| Turdus migratorius | 77.36 | 3.73 | 12.92 | Bennett PM 2002 |
| Zenaida macroura | 119.76 | 2 | 14.8 | Bennett PM 2002 |
